# Mitochondrial Transplantation Attenuates Cerebral Ischemia-Reperfusion Injury: Possible Involvement of Mitochondrial Component Separation

**DOI:** 10.1155/2021/1006636

**Published:** 2021-11-20

**Authors:** Qiang Xie, Jun Zeng, Yongtao Zheng, Tianwen Li, Junwei Ren, Kezhu Chen, Quan Zhang, Rong Xie, Feng Xu, Jianhong Zhu

**Affiliations:** Fudan University Huashan Hospital, Department of Neurosurgery, National Center for Neurological Disorders, National Key Laboratory for Medical Neurobiology, Shanghai Key Laboratory of Brain Function and Regeneration, Institutes of Brain Science, MOE Frontiers Center for Brain Science, Shanghai Medical College-Fudan University, 12 Wulumuqi Zhong Rd., Shanghai 200040, China

## Abstract

**Background:**

Mitochondrial dysfunctions play a pivotal role in cerebral ischemia-reperfusion (I/R) injury. Although mitochondrial transplantation has been recently explored for the treatment of cerebral I/R injury, the underlying mechanisms and fate of transplanted mitochondria are still poorly understood.

**Methods:**

Mitochondrial morphology and function were assessed by fluorescent staining, electron microscopy, JC-1, PCR, mitochondrial stress testing, and metabolomics. Therapeutic effects of mitochondria were evaluated by cell viability, reactive oxygen species (ROS), and apoptosis levels in a cellular hypoxia-reoxygenation model. Rat middle cerebral artery occlusion model was applied to assess the mitochondrial therapy in vivo. Transcriptomics was performed to explore the underlying mechanisms. Mitochondrial fate tracking was implemented by a variety of fluorescent labeling methods.

**Results:**

Neuro-2a (N2a) cell-derived mitochondria had higher mitochondrial membrane potential, more active oxidative respiration capacity, and less mitochondrial DNA copy number. Exogenous mitochondrial transplantation increased cellular viability in an oxygen-dependent manner, decreased ROS and apoptosis levels, improved neurobehavioral deficits, and reduced infarct size. Transcriptomic data showed that the differential gene enrichment pathways are associated with metabolism, especially lipid metabolism. Mitochondrial tracking indicated specific parts of the exogenous mitochondria fused with the mitochondria of the host cell, and others were incorporated into lysosomes. This process occurred at the beginning of internalization and its efficiency is related to intercellular connection.

**Conclusions:**

Mitochondrial transplantation may attenuate cerebral I/R injury. The mechanism may be related to mitochondrial component separation, altering cellular metabolism, reducing ROS, and apoptosis in an oxygen-dependent manner. The way of isolated mitochondrial transfer into the cell may be related to intercellular connection.

## 1. Introduction

Stroke is an acute cerebrovascular disease, including ischemic and hemorrhagic stroke, and is considered to be one of the leading causes of human death and disability worldwide [1–4]. Ischemic stroke accounts for over 80% of all strokes and is usually triggered by brain arterial embolism [3, 5]. When blood flow is blocked, brain tissue in the area of blood supply becomes ischemic and hypoxic, which then leads to neurological dysfunction. Moreover, following blood reperfusion, the damaged brain tissue can be further harmed by restoration of oxygen-rich blood, causing a so-called ischemia-reperfusion (I/R) injury [3, 6–9]. Major contributors to the pathological process include overproduction of ROS, dramatically increased extracellular glutamate levels, and activation of neuroinflammation responses [6, 7, 9]. Among these, the dysfunction of mitochondria in neurons plays a pivotal role [3, 9]. Currently, the main strategy to mitigate ischemic stroke injury is revascularization [4, 8, 10], which may lead to I/R injury. Despite remarkable progress that has been achieved for ischemic stroke, there seem to be no better options for I/R injury.

Studies have shown that mitochondria are not only the energy factories of cells but are also closely related to other biological processes, including calcium homeostasis, ROS production, hormone biosynthesis, and cellular differentiation [3, 9, 11]. Mitochondria play an important role in many diseases. Recently, a growing number of studies have begun to apply isolated mitochondria as a therapeutic agent to treat diseases, including kinds of I/R injury [12–20], liver disorders [21, 22], breast cancer [23–25], lung diseases [26, 27], and central nervous system disorders [28–37]. Furthermore, Emani et al. conducted an autologous mitochondrial transplantation clinical study, which showed a promising clinical application [38, 39]. Also, there are studies registered at ClinicalTrials.gov (NCT03639506, NCT02851758, and NCT04998357). Therefore, mitochondrial transplantation holds great therapeutic potential for cerebral I/R injury. One big concern of mitochondrial transplantation is an immune and inflammatory response based on data of mtDNA [40] and damage-associated molecular patterns (DAMPs) [41]. Ramirez-Barbieri et al. demonstrated that there is no direct or indirect, acute or chronic alloreactivity, allorecognition, or DAMP reaction to single or serial injections of allogeneic mitochondria [42].

Recently, several studies have applied isolated mitochondria from various sources as an intervention in many diseases. Four studies focused on cerebral I/R injury have shown benefits of mitochondrial transplantation based on various phenotypes, such as behavioral assessment, infarct size, ROS, and apoptosis. However, the appropriate source of mitochondria, the mechanism of its therapeutic effect, and the fate of isolated mitochondria remain unclear. The clinical application of isolated mitochondria has just begun, and more safety and effectiveness assessments are needed.

In order to answer the above questions, we performed this study. Firstly, we evaluated the source of mitochondria and then assessed the therapeutic effects of mitochondrial transplantation in cellular and animal models. Finally, we mainly focused on the therapeutic mechanisms of mitochondrial transplantation and the fate of transplanted mitochondria.

## 2. Methods

### 2.1. Cells

The mouse neural stem cell (mNSC) was obtained and cultured as previously described [43]. Adherent culture of mNSC was performed with Matrigel (Corning, NY, USA, Cat#354277). N2a (Cat#SCSP-5035) and induced pluripotent stem cell (iPSC) (Cat#DYR0100) were purchased from the National Collection of Authenticated Cell Cultures, Shanghai. 293T was provided by Dr. Gao Liu from Zhongshan Hospital, Shanghai Medical College, Fudan University. N2a and 293T cells were cultured in DMEM supplemented with 10% fetal bovine serum. The iPSC was cultured according to the manufacturer's protocol.

### 2.2. Animals

Sprague-Dawley rats (7-8 weeks old, 250-300 g) were obtained from Shanghai Super-B&K Laboratory Animal Corp. Ltd. (Shanghai, China). All experimental procedures and animal care were approved by the Animal Welfare and Ethics Group, Laboratory Animal Science Department, Fudan University (ethical approval number 202006013Z) and were carried out according to the Guidelines for the Care and Use of Laboratory Animals by the National Institutes of Health. The rats were divided into three groups: sham, I/R, and I/R+Mito group (sham = sham-operated; I/R = MCAO+reperfusion with saline injection; I/R+Mito = MCAO+reperfusion with mitochondria injection).

### 2.3. Mitochondrial Isolation

Mitochondria were isolated from N2a and mNSC using the mitochondria isolation kit (ThermoFisher Scientific, USA, Cat#89874) as previously described [32, 44]. Briefly, after cultured cells were orderly digested (trypsin) and centrifuged (300 g, 5 min) and the supernatant was removed, collected cells were resuspended by mitochondrial isolation reagent A (800 *μ*l) in a 2.0 ml microcentrifuge tube and vortexed for 5 s and then incubated for 2 min on ice. Then, the reagent B (10 *μ*l) was further added into the tube and continuously placed in situ for 5 min. Following vortexed at maximal speed for 5 times (each time for 1 min), the reagent C (800 *μ*l) was added into the tube and mixed. Subsequently, the mixed solution was centrifuged (700 g, 10 min, 4°C) and then the supernatant was obtained for further centrifugation (12000 g, 15 min, 4°C). Finally, fresh mitochondria were obtained and used for further experiments. For animal experiments, each rat received mitochondria isolated from 1 × 10^7^ cells, and the protein content was about 180 *μ*g-200 *μ*g.

### 2.4. Transmission Electron Microscopy (TEM)

Cells were fixed with 2.5% glutaraldehyde for 2 h at room temperature and then centrifuged (300×g, 5 min). Subsequently, cells were postfixed with precooled 1% osmic acid (2 h, 4°C) and then centrifuged again (300×g, 5 min). After gradient alcohol dehydration and penetration with a solution of acetone and epoxy resin at different proportions, the cell samples were further embedded into epoxy resin and solidified for 48 h. Subsequently, the embedded samples were sectioned (thickness: 60-100 nm) and then double-stained with 3% uranyl acetate and lead citrate. Finally, the stained sections were observed and imaged by TEM (Tecnai G2 20 TWIN, FEI Company, Oregon, USA).

### 2.5. Mitochondrial Membrane Potential Analysis

The mitochondrial membrane potential (MMP/*ΔΨ*m) was assessed by JC-1 dye (Beyotime Biotechnology, Shanghai, China, Cat#C2006) and detected by flow cytometry and confocal microscopy, according to previous methods [45, 46]. For flow cytometry, single-cell suspensions of mNSC and N2a were prepared and then coincubated with JC-1 work solution for 20 min at 37°C. Next, sample cells were centrifuged (600 g, 4°C, 5 min) and washed with JC-1 buffer solution 2 times. Subsequently, resuspended cells were subjected to flow cytometry tests. For image, cells were seeded in glass-bottom Petri dishes. 24 hours later, cells were coincubated with JC-1 work solution for 20 min at 37°C, washed with JC-1 buffer, and then examined by confocal microscopy.

### 2.6. Polymerase Chain Reaction (PCR)

Absolute quantitative PCR was performed as previously described [47–49]. The ratio of mtDNA and nuclear DNA was used to assess relative mtDNA copy number. In this experiment, mt-ND1/*β*-globin and mt-RNR1/*β*-actin were used to represent abundance. The sequences of the primers are described in Table S1.

### 2.7. Mitochondrial Stress Test

A mitochondrial stress test was performed using the Seahorse XF Cell Mito Stress Test Kit according to the manufacturer's instruction [33, 49, 50]. Oxygen consumption rate (OCR), basic OCR, and maximal OCR were used as the main evaluation indicators. Different levels of cells were tested, including 1 × 10^5^ and 2 × 10^5^.

### 2.8. Hypoxia-Reoxygenation (H/R) Cell Model and Mitochondrial Transplantation

The H/R cell model was induced by 48 h of hypoxia (1% O_2_) in a tri-gas CO_2_ incubator and 24 h of routine culture, according to previously described methods [51]. The cultured cells were divided into 3 groups: control group (routine culture (48 h)+replacing medium+routine culture (24 h)), H/R group (hypoxic culture (48 h)+replacing medium+continuing routine culture (24 h)), and H/R+mitochondrial treatment group (hypoxic culture (48 h)+ replacing medium (containing exogenous mitochondria)+continuing routine culture (24 h)). The ratio of mitochondrial donor cell number to the receiver is 5 (e.g., 2 × 10^5^ cells need mitochondria isolated from 1 × 10^6^ cells).

### 2.9. Cell Viability Assay

The viability was assessed by Cell Counting Kit-8 (CCK-8) (Dojindo Laboratories, Kumamoto, Japan, Cat#CK04) according to the manufacturer's instruction. Briefly, N2a were coincubated with the CCK-8 working solution at 37°C for 3 h in the light-avoided environment. Then, cells were detected at 450 nm by a microplate reader (Molecular Devices, Sunnyvale, CA, USA).

### 2.10. ROS Measurement by Flow Cytometry

DCFH-DA probes (Beyotime Biotechnology, Shanghai, China, Cat#S0033S) were used to measure the ROS levels in cells according to the manufacturer's instruction. Fluorescence intensity was detected by flow cytometry and fluorescence plate reader. Briefly, after coincubated with DCFH-DA probes (10 *μ*mol/l, excitation wavelength: 488 nm and emission wavelength: 525 nm) at 37°C for 30 min, cells were detected by a microplate reader (Molecular Devices, Sunnyvale, CA, USA) or collected by centrifugation (300 g, 5 min); after resuspended with PBS, DCFH-DA-labeled cells were further detected by flow cytometry.

### 2.11. Western Blot

Western blot was performed as previously described [52, 53]. The following primary antibodies were used for WB detection: anti-MFN1 (1 : 500), anti-OPA1 (1 : 1000), and anti-DRP1 antibodies (1 : 1000) were all purchased from Proteintech (Chicago, IL, USA); and anti-Bax (1 : 2000), anti-Bcl-2 (1 : 2000), anti-caspase-3 (1 : 2000), and anti-GAPDH antibodies (1 : 10000) were all purchased from Abcam (Cambridge, Cambs, UK). GAPDH served as internal reference. WB bands were detected with Gel-Pro Analyzer (Media Cybernetics, MD, USA).

### 2.12. Cell Apoptosis

Cell apoptosis was evaluated using an Annexin V-FITC/PI Apoptosis Detection Kit (BD Biosciences, NJ, USA, Cat#40302) according to the manufacturer's instruction. Briefly, cells were coincubated with Annexin V-FITC and then propidium iodide for 15 min at RT in a light-avoided environment and then detected by flow cytometry.

### 2.13. Middle Cerebral Artery Occlusion (MCAO)

Intraluminal filament occlusion was used to induce focal cerebral ischemia injury [54, 55]. Anesthetized by 2% pentobarbital sodium (45 mg/kg), the rats were placed in a prone position. Then, the left common carotid artery, external carotid artery (ECA), and internal carotid artery (ICA) were exposed. Next, a silicon-coated monofilament suture was gradually inserted through the left ECA and was moved up into the left ICA to successfully occlude the left middle cerebral artery (MCA) and remained in situ for 120 min. Subsequently, the suture was carefully removed, the ECA was permanently ligated, and the incision was sutured. Sham group rats were subjected to the same procedure except for the 120 min occlusion of MCA. Experimental animals were then placed into individual cages and provided a standard diet and water. After 120 min occlusion, right before ICA reperfusion, the isolated mitochondria (from 1 × 10^7^ cells, the protein content was about 180 *μ*g-200 *μ*g) or saline (10 *μ*l) was injected into the ICA and all incisions were closed.

### 2.14. Neurobehavioral Evaluation

Neurobehavioral deficits were evaluated 24 h after mitochondrial transplantation using multiple scales, including the Clark general functional deficit score [56, 57], the Clark focal functional deficit score [56, 57], the modified neurological severity score (mNSS) [55, 58], and the rotarod test [55, 59]. Behavioral assessments were conducted by two skillful investigators who were both blinded to the animal groups.

### 2.15. Cerebral Infarct Area Detection

Triphenyl tetrazolium chloride (TTC) staining was used to display the area of cerebral infarction [60, 61]. Briefly, 24 h after MCAO, the rats were deeply anesthetized and perfused with PBS transcardially, after which the rat brains were obtained and cut into 2 mm thick coronal sections. Subsequently, the brain sections were incubated with a 2% TTC solution at 37°C for 30 min in darkness. Then, stained slices were placed from the frontal to occipital order, and macroscopic images were obtained with a digital camera. Infarct areas were measured by Adobe Photoshop 21.0.0 (Adobe Systems Inc., San Jose, CA, USA).

### 2.16. Transcriptomic Analysis

RNA sequencing was performed as previously described [62, 63]. Downstream analysis was performed by R (R Foundation for Statistical Computing, Vienna, Austria).

### 2.17. Mitochondria and Lysosome Labeling

The mitochondrial fluorescent dyes MitoTracker™ Red CMXRos (ThermoFisher Scientific, Waltham, MA, USA), MitoTracker™ Green FM (ThermoFisher Scientific, Waltham, MA, USA), and MitoBright Deep Red (Dojindo Laboratories, Kumamoto, Japan) were used to label mitochondria according to the manufacturer's instruction. In addition, 293T cells expressing COX8A gene N-terminal signal peptide-mCherry fusion protein were constructed by lentivirus (Inovogen Tech, Chongqi, China, Cat#3512) and the mitochondria were well labeled. The Lyso Dye (Dojindo Laboratories, Kumamoto, Japan, Cat#MD01)was used to label lysosomes according to the manufacturer's instruction.

### 2.18. Statistical Analysis

Data that conform to a normal distribution with homogeneous variance are expressed as mean ± standard deviation (SD), and Student's *t*-test or one-way analysis of variance (ANOVA) was used to compare the differences between two groups or among multiple groups, respectively. Data with a nonnormal distribution are presented as median (25%, 75% quantiles), and Mann-Whitney *U*-test was taken into consideration. Statistical analysis and diagram generation were performed using GraphPad Prism 8.0.1 (GraphPad Software, Inc., San Diego, CA, USA). ^∗^*p* < 0.05 and ^∗∗^*p* < 0.01 were considered to be statistically different.

## 3. Results

### 3.1. Characteristics of Mitochondrial Donors

**Figure 1:**
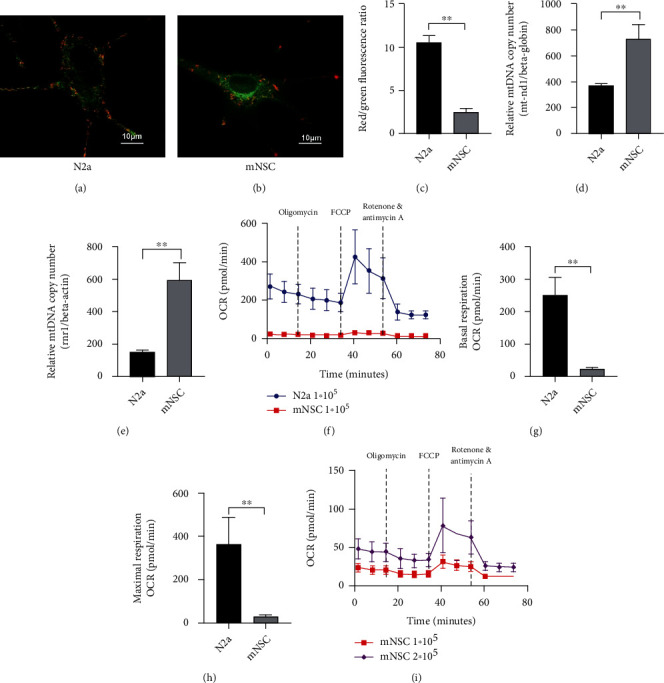
Characteristics of mitochondrial donors. (a–c) Mitochondria of N2a (a) and mNSC (b) labeled with JC-1, JC-1 aggregates show red and monomers show green; the fluorescence intensity ratio of JC-1 of N2a and mNSC detected by flow cytometry, N2a showed higher *ΔΨ*m ((c), *n* = 3). (d, e) Relative mtDNA copy number of N2a and mNSC indicated by mt-ND1/*β*-globin (d) and RNR1/*β*-actin (e), N2a showed lower mtDNA copy number (*n* = 3). (f–i) Seahorse XF cell mitochondrial stress test; the performance of N2a and mNSC at 1 × 10^5^ level (f), N2a exhibited higher oxidative respiratory activity (*n* = 6); quantitative analysis of basal (g) and maximal (h) OCR of two cells showed that N2a exhibits higher oxidative respiratory activity (*n* = 9). The performance of mitochondrial stress test form NSC at 1 × 10^5^ (same data as in (f)) and 2 × 10^5^ level showed mNSC reacted well and did not die in the comparison with N2a (i); we did the test with N2a 1 × 10^5^, mNSC 1 × 10^5^, and mNSC 2 × 10^5^ at one time but presented them in two graphs due to the order of magnitude. ^∗∗^*p* < 0.01.

The ideal source of mitochondria is one that is readily available and can be amplified in large numbers. Both stem cells and tumor cells meet this requirement. Therefore, we chose N2a and mNSC as mitochondrial source cells to assess a range of mitochondrial characteristics. To evaluate the *ΔΨ*m, JC-1 dye was used. Representative images showed N2a has more red components than mNSC (Figures 1(a) and 1(b)). Flow cytometry analysis confirmed that N2a had a higher *ΔΨ*m than mNSC (N2a vs. mNSC: 10.55 ± 0.85 vs. 2.56 ± 0.36, *p* < 0.01) (Figure 1(c)). In addition, we observed that the mtDNA abundance of mNSC was higher than N2a based on the mitochondrial-nuclear DNA ratio, mt-ND1/*β*-globin (N2a vs. mNSC: 374.0 ± 11.5 vs. 731.1 ± 110.4, *p* < 0.01) (Figure 1(d)) and mt-RNR1/*β*-actin (N2a vs. mNSC: 149.1 ± 13.07 vs. 593.4 ± 108.3, *p* < 0.01) (Figure 1(e)). We subsequently analyzed the oxidative respiration capacity of mitochondria from N2a and mNSC based on the Seahorse XF analysis platform. The OCR-time diagram is shown in Figure 1(f) (N2a 1∗10^5^ vs. mNSC 1∗10^5^) and Figure 1(i) (mNSC 1∗10^5^ vs. mNSC 2∗10^5^), which implied a huge difference in oxidative respiratory activity between N2a and mNSC. Basal OCR of N2a (1 × 10^5^ cells) was significantly higher than those of mNSC (1 × 10^5^ cells) (N2a vs. mNSC: 248.70 ± 56.33 pmol/min vs. 22.14 ± 5.09 pmol/min, *p* < 0.01) (Figure 1(g)). Similarly, N2a (1 × 10^5^ cells) exhibited higher maximal OCR values than those of mNSC (1 × 10^5^ cells) (N2a vs. mNSC: 363.90 ± 123.70 pmol/min vs. 28.14 ± 7.50 pmol/min, *p* < 0.01) (Figure 1(h)). These results suggested that compared to mNSC, mitochondria from N2a exhibited a relatively stronger oxidative respiration capacity. In addition, mitochondrial morphology is presented in Figure S1 and mNSC culture and identification data are presented in Figure S2. Metabolic profiles of N2a and mNSC were quite different (Figure S3). Tumorigenicity evaluation of N2a and mNSC is presented in Figure S4. These results suggested that N2a-derived mitochondria have higher oxidative respiratory activity and lower mtDNA copy number, that the mitochondria from mNSC and N2a have similar morphology, and that they have different metabolomic profiles, and neither is tumorigenic. Therefore, we chose the N2a as a major source of mitochondria for subsequent experiments.

### 3.2. Mitochondrial Transplantation Increased Cell Viability and Attenuated ROS and Apoptosis Level under H/R Condition

**Figure 2:**
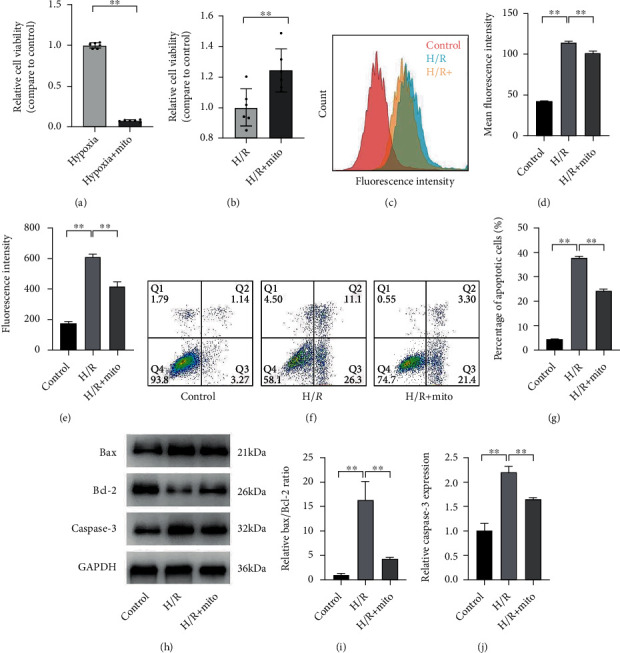
Mitochondrial transplantation increased cell viability and attenuated ROS and apoptosis level under H/R condition. (a, b) Cell viability measured by CCK-8; after 48 h of hypoxia, exogenous mitochondria were added and N2a continued to be cultured in a hypoxic (a) or normoxic (b) environment (*n* = 6). (c–e) ROS levels labeled with DCFH-DA probes and measured by flow cytometry, presented as a typical histogram of fluorescence intensity distribution (c) and a fluorescence intensity bar graph ((d), *n* = 3), and fluorescent plate reader ((e), *n* = 3). (f–j) Apoptosis levels were detected by flow cytometry for Annexin V and PI positivity ((f, g), *n* = 3) and by related protein expression; representative WB bands were obtained (h), and correspondingly, quantitative analysis of Bax/Bcl-2 (i) and caspase-3 (j) was shown. Values were reported as means ± SD. ^∗∗^*p* < 0.01.

We verified the effect of exogenous mitochondria on cell viability in a cellular model. After 48 h of hypoxia (1% O_2_), exogenous mitochondria were added and N2a continued being cultured for the next 24 h, and then, CCK-8 was performed to detect cell viability. The result indicated that the cell viability was correlated with the presence of oxygen after mitochondrial intervention. When mitochondrial-treated N2a continued being cultured under a hypoxic condition, the cell viability decreased dramatically (hypoxia vs. hypoxia+Mito: 1.00 ± 0.03 vs. 0.07 ± 0.01, *p* < 0.01) (Figure 2(a)). When continued being cultured under a reoxygenation condition, the exogenous mitochondrial intervention significantly improved the cell viability (reoxygenation vs. reoxygenation+Mito: 1.00 ± 0.12 vs. 1.24 ± 0.14, *p* < 0.01) (Figure 2(b)). To evaluate the ROS levels, DCFH-DA probes were applied and the fluorescence intensity was measured by flow cytometry and fluorescence plate reader. The flow cytometry results showed the H/R intervention significantly increases ROS levels and exogenous mitochondrial transplantation attenuates that process (control, H/R, H/R+Mito: 42.6 ± 0.17, 115.0 ± 1.00, and 101.7 ± 2.41, *p* < 0.05) (Figures 2(c) and 2(d)). Similarly, the fluorescent plate reader confirmed the results (control, H/R, H/R+Mito: 173.2 ± 13.74, 606.1 ± 23.45, and 416.9 ± 31.59, *p* < 0.01) (Figure 2(e)). To measure the apoptosis level, we performed flow cytometry analysis and Western blot. After H/R injury, the apoptosis ratio of N2a dramatically increased (H/R vs. control: 37.90 ± 0.46% vs. 4.44 ± 0.07%, *p* < 0.01) (Figures 2(f) and 2(g)), which was significantly reduced by mitochondrial transplantation (H/R vs. Mito+H/R: vs. 24.35 ± 0.54%, *p* < 0.01) (Figures 2(f) and 2(g)). Similar results were obtained for the expression levels of apoptosis-related proteins, which also suggested that H/R dramatically promoted the upregulation of the Bax/Bcl-2 ratio (H/R vs. control: 16.28 ± 3.82 vs. 1.00 ± 0.11, *p* < 0.01) (Figures 2(h) and 2(i)) and caspase-3 protein (H/R vs. control: 2.21 ± 0.12 vs. 1.00 ± 0.16, *p* < 0.01) (Figures 2(h) and 2(j)). Exogenous mitochondrial transplantation significantly downregulated the ratio of Bax/Bcl-2 (H/R vs. H/R+Mito: vs. 4.25 ± 0.34, *p* < 0.01) (Figures 2(h) and 2(i)) and protein levels of caspase-3 (H/R vs. H/R+Mito: vs. 1.65 ± 0.03, *p* < 0.01) (Figures 2(h) and 2(j)) in cultured cells.

### 3.3. Mitochondrial Transplantation Improved Neurobehavioral Deficits and Reduced Infract Size of MCAO Rats

**Figure 3:**
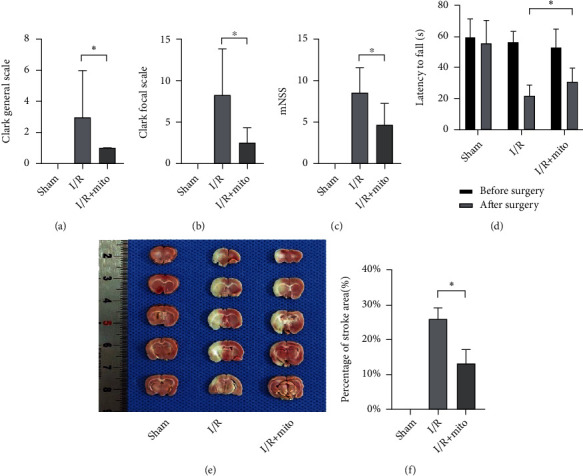
Mitochondrial transplantation improved neurobehavioral deficits and reduced infarct size of MCAO rats. (a–d) Neurological behavior assessment; 24 h after mitochondrial transplantation, multiple score scales were used to evaluate the neurological deficits induced by MCAO, including Clark general functional deficit score (a), Clark focal functional deficit score (b), mNSS score (c), and rotarod test (d). (e, f) Infarction size evaluation; brain infarction areas were stained by TTC (e), and relatively quantitative analysis of infarction size was assessed (f). Values were reported as means ± SD. ^∗^*p* < 0.05. Sham = sham-operated; I/R = MCAO+reperfusion with saline injection; I/R+Mito = MCAO+reperfusion with mitochondrial injection.

To evaluate the effect of exogenous mitochondria on behavior and infarct size, we used the MCAO model. The Clark general/focal scale, the mNSS, and the rotarod test were used to assess neurological behavior deficits, and TTC staining was performed to measure infarct size. The results suggested that mitochondrial transplantation significantly improved neurological behavior deficits. For sham, I/R, and I/R+Mito groups, the sample size was 8, 9, and 7, and the Clark general scale was 0 (0,0), 3 (1.5,6), and 1 (0,1) (nonnormally distributed data are expressed as median, 25%, 75% quantile), which indicated that mitochondria improved neurological outcome (*p* < 0.05). The Clark focal scale and mNSS confirmed the following: Clark focal: 0 ± 0, 8.33 ± 5.57, and 2.57 ± 1.81, *p* < 0.05; mNSS: 0 ± 0, 8.56 ± 3.01, and 4.71 ± 2.63, *p* < 0.05) (Figures 3(a)–3(c)). The rotarod test also validated the conclusion. The latency time to fall of sham, I/R, and I/R+Mito groups before the surgery was 59.63 ± 11.77 s, 56.33 ± 6.76 s, and 53.43 ± 11.66 s (*p* > 0.05) and 55.63 ± 15.01 s, 21.78 ± 6.78 s, and 30.71 ± 8.98 s (*p* < 0.05) after the surgery (Figure 3(d)). The TTC results suggested exogenous mitochondrial transplantation could reduce the infarct size of the MCAO model (I/R vs. I/R+Mito 26.02 ± 3.24% vs. 13.36 ± 4.00%, *p* < 0.05) (Figures 3(e) and 3(f)).

### 3.4. Effects of Mitochondrial Transplantation on Transcriptomic Profile Imply Metabolic Alteration

**Figure 4:**
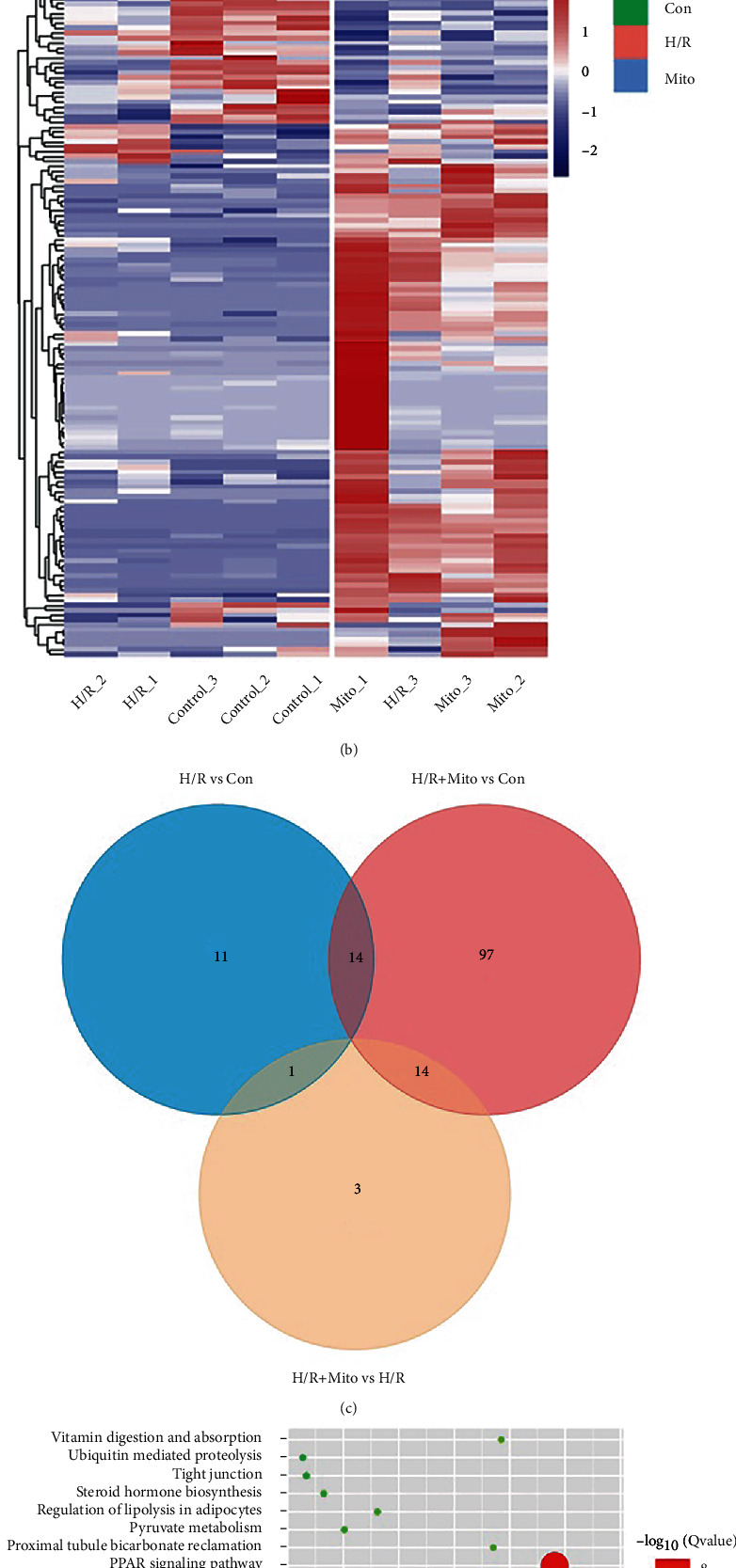
Effects of mitochondrial transplantation on transcriptomic profile imply metabolic alteration. (a) PCA plot, axis 1 18.6%, axis 2 16.5%. (b) Heatmap, horizontal axis-different cell samples; longitudinal axis-different genes; color depth-expression levels of genes. (c) Venn diagram. (d) KEGG bubble chart of the differentially expressed genes between H/R and H/R+Mito groups.

In order to clarify the effects of mitochondrial transplantation on transcriptomic profile and its possible mechanism, we performed RNA sequence analysis. The results identified 14 upregulated genes and 12 downregulated genes between control and H/R groups, 27 upregulated genes and 98 downregulated genes between control and H/R+Mito groups, and 17 upregulated genes and 1 downregulated gene between H/R and H/R+Mito groups. The overlapping genes are presented in Figure 4(c). The following KEGG pathway enrichment analysis suggested exogenous mitochondria may affect metabolism-related pathways, especially lipid metabolism-related molecules and pathways such as the PPAR signal pathway, insulin signal pathway, fat intake and digestion-related pathway, cholesterol metabolism, glycolysis, and gluconeogenesis (Figure 4(d)). These results indicated that exogenous mitochondria may be capable of altering the metabolic characteristics of host cells, possibly resulting in metabolic reprogramming.

### 3.5. Pattern of Exogenous Mitochondrial Transfer Implies Mitochondrial Component Separation

**Figure 5:**
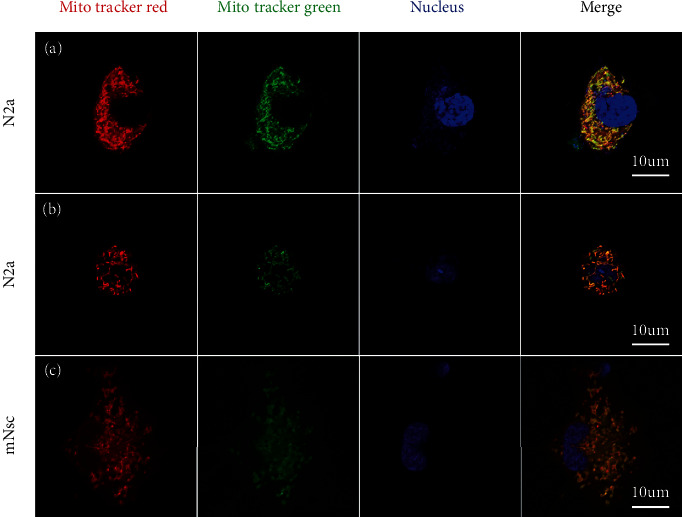
Exogenous mitochondria colocalized with endogenous mitochondria despite species by coincubation. (a) From left to right, mitochondria of host N2a labeled with MitoTracker Red; isolated green mitochondria entered host N2a; nucleus with DAPI; the merged image. (b) Isolated red mitochondria entered host N2A; mitochondria of host N2a labeled with MitoTracker Green; DAPI; the merged image. (c) Isolated red mitochondria derived from U87 entered host mNSC; mitochondria of host mNSC labeled with MitoTracker Green; DAPI: the merged image. Scale bar: 10 *μ*m.

To better understand the mechanism of exogenous mitochondria, we first traced its pathway. MitoTracker™ Red CMXRos and MitoTracker™ Green FM were used to label mitochondria. When the mitochondria of N2a labeled with MitoTracker Green were isolated and added to the medium of another N2a which was labeled with MitoTracker Red, a few hours later, we found that the red and green were completely fused (Figure 5(a)). This phenomenon implied that exogenous mitochondria can be fused with endogenous mitochondria of the host cell. Next, we isolated red dye-labeled mitochondria and added them to the medium of green dye-labeled cell. The result validated the previous (Figure 5(b)). To figure out if this fusion property of exogenous mitochondria is species-limited, we isolated red dye-labeled mitochondria from a human-derived cell line-U87 and added it to mNSC medium. Mitochondria fused again (Figure 5(c)). This suggested that the ability of exogenous mitochondria to fuse with host cell mitochondria is cross-species.

**Figure 6:**
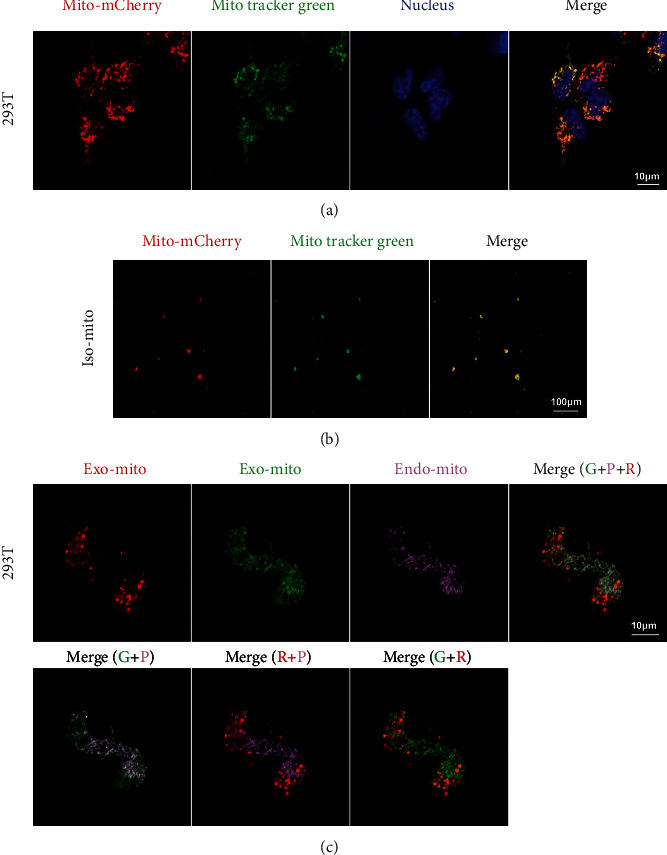
Double-labeled exogenous mitochondria exhibited component separation after being internalized by host cells. (a) Double-labeled mitochondria of 293T; from left to right, mitochondria labeled with a fluorescent protein (Mito-mCherry); mitochondria labeled with MitoTracker Green; DAPI; the merged image showed completely colocalization. (b) Isolated mitochondria labeled with red and green markers; from left to right, isolated mitochondria labeled with Mito-mCherry; isolated mitochondria labeled with MitoTracker Green; the merge image showed well co-localization. (c) From left to right, top to bottom; the red components of exogenous mitochondria entered the host cell, and the distribution was shown; the green components of exogenous mitochondria entered the host cell and its distribution; endogenous mitochondria of the host cell labeled with MitoBright Deep Red (pink); the merged image of three colors showed fusion and separation; the merged image of green and pink indicated that parts of the green overlap with the pink; the merged image of red and pink showed that seldom red components overlap with the pink; the merged image of green and red suggested that a portion of red components overlaps with the green components. Scale bar: 10 *μ*m, 100 *μ*m, and 10 *μ*m.

**Figure 7:**
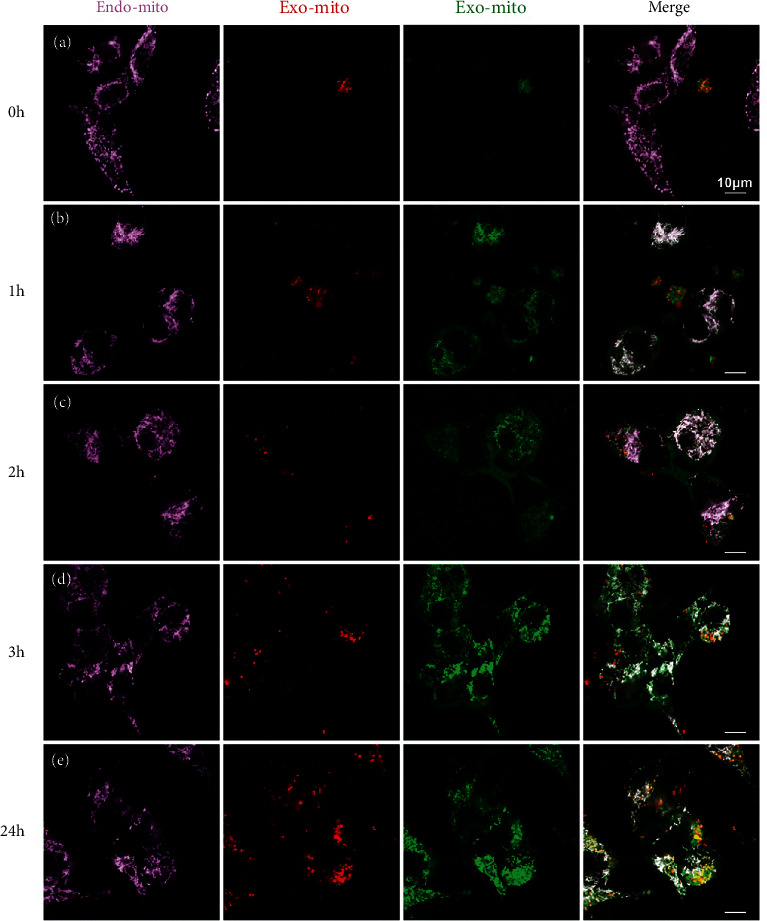
Time pattern of different mitochondrial components during transfer. (a) Mitochondria of host 293T cell labeled with MitoBright Deep Red; isolated double-labeled mitochondria were added to the medium of host cell instantly. (b) 1 h after coincubation. (c) 2 h after coincubation. (d) 3 h after coincubation. (e) 24 h after coincubation. Scale bar: 10 *μ*m.

**Figure 8:**
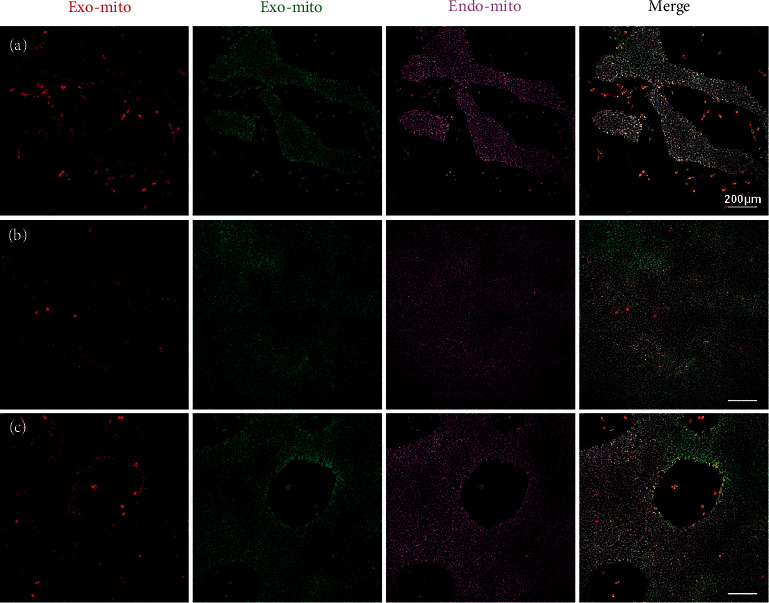
Intercellular connections affect the transfer of different components of mitochondria. (a) Small iPSC clone; mitochondria of host iPSC labeled with MitoBright Deep Red (Endo-Mito); isolated double-labeled mitochondria (Exo-Mito) were added to the medium of host cell; the picture showed the green components of exogenous mitochondria colocalized with endogenous mitochondria of host iPSC, while the red components mainly concentrated at the edges of cell clones and scattered cells. (b) iPSC clone with tight intercellular connections; the red component entered the cell in a random pattern. (c) iPSC clone with tight intercellular connections and edges; the red component mainly concentrated at the edges of cell clones and scattered cells. Scale bar: 200 *μ*m.

To further verify the fusion property of exogenous mitochondria, we constructed a 293T cell overexpressing COX8A N-terminal signal peptide-mCherry to labeled mitochondria. And this tag had little effect on cell and mitochondrial function (Figure S5). When the red-mitochondria 293T cell was labeled with MitoTracker Green again, we found all the mitochondria have both red and green markers (Figure 6(a)). The mitochondria isolated from the double-labeled cell also showed the colocation of the two colors (Figure 6(b)). When we added the double-labeled isolated mitochondria to the medium of a 293T cell labeled with MitoBright Deep Red (set to pink), we came to an interesting result. The pink mitochondria (endogenous mitochondria of host cell) completely colocalized with exogenous green mitochondria, while only a portion of pink mitochondria overlapped with exogenous red mitochondria. Moreover, the two-color marker of the same exogenous mitochondria was partially separated (Figure 6(c)). All of these suggested that the exogenous mitochondrial components segregate when cocultured with host cells and a specific part can fuse with the endogenous mitochondria of host cells and the rest part has another fate. During the mitochondrial transfer process, we found that the green component can be internalized immediately by the host cell within 1 h, while the red component was internalized in a much slower way (Figures 7(a)–7(e)). This again confirmed the different fate of different mitochondrial components. We further investigated the pattern of exogenous mitochondrial transfer using double-labeled 293T as a mitochondrial donor and pink-labeled induced pluripotent stem cell (iPSC) as host. The results showed nearly all the green components colocalized with the pink endogenous mitochondria and the red component was concentrated at the edge of the cell clones or in the scattered cells (Figures 8(a)–8(c)). These suggested the internalization efficiency of red component may be related to intercellular connections.

### 3.6. The Fate of Exogenous Mitochondria Is Fusion and Lysosomal Degradation

**Figure 9:**
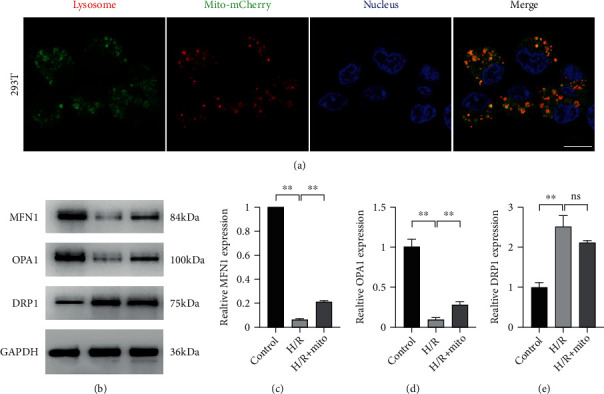
The fate of exogenous mitochondria is fusion and lysosomal degradation. (a) Endogenous lysosomes were marked by Lyso Dye (green), and exogenous mitochondria were labeled with COX8A N-terminal signal peptide-mCherry (red); the red mitochondria colocalized with green lysosomes. (b–e) WB analysis of mitochondrial dynamic proteins; typical WB bands of MFN1, OPA1, and DRP1 proteins were obtained (b), and relatively quantitative analysis of MFN1 (c), OPA1 (d), and DRP1 (e) was carried out. Scale bar: 10 *μ*m. Values were reported as means ± SD. ^∗∗^*p* < 0.01; ns: not statistical significant, *p* > 0.05.

To prove the theory that a portion of exogenous mitochondria can fuse with endogenous mitochondria, we assessed the mitochondrial dynamics by WB analysis. Our results suggested that after H/R treatment, the expression levels of the mitochondrial fusion-related proteins MFN1 (control vs. H/R, *p* < 0.01) and OPA1 (control vs. H/R, *p* < 0.01) were dramatically reduced and mitochondrial fission-related protein DRP1 was significantly increased (control vs. H/R, *p* < 0.01). Exogenous mitochondrial intervention alleviated this process and increased the expression of MFN1 (H/R vs. H/R+Mito, *p* < 0.01) and OPA1 (H/R vs. H/R+Mito, *p* < 0.01) but did not significantly reverse DRP1 expression (Figures 9(b)–9(e)). The above data further confirm the fusion property of exogenous mitochondria.

To figure out the fate of the unfused, COX8A N-terminal signal peptide-mCherry fusion protein-labeled mitochondria, we did lysosomal staining with Lyso Dye. Interestingly, the red component of mitochondria is totally colocalized with lysosomes of the host cell (Figure 9(a)), suggesting the fate of unfused components of exogenous mitochondria is lysosomal degradation.

## 4. Discussion

There are four studies [30, 31, 64, 65] focused on the treatment of cerebral I/R injury with isolated mitochondria, according to the latest review [66]. All of them showed a favorable outcome in behavioral assessment or cerebral infarct size with different mitochondrial donors by intravenous or intracerebroventricular injection. However, there is no more detailed information about the source of mitochondria, the mechanism, and the fate of isolated mitochondria. And this is the information we want to provide.

In order to apply mitochondrial transplantation therapy to the clinic, the first priority is the source and quality control of mitochondria. The ideal source of mitochondria is one that is readily available and can be amplified in large numbers. Both stem cells and tumor cells meet this requirement. Therefore, we chose N2a and mNSC as mitochondrial source cells to assess a range of mitochondrial characteristics. Previous studies isolated mitochondria from the placenta [65], or human umbilical cord-derived mesenchymal stem cells [64], or pectoralis major muscle [30], or baby hamster kidney fibroblast [31], and evaluated them mainly by MMP or respiratory activity. The outcome is closely related to the isolation and preservation process, and the consistency may not be good among different batches. The four studies did not give the reason why they choose these cells as mitochondrial donors. However, in our study, we evaluated the mitochondria before isolation through multiple dimensions, including morphology, MMP, mtDNA copy number, respiratory activity, metabolomic profile, and tumorigenicity. We hope to provide reference data when choosing a mitochondrial donor in future research.

For the first time, we have identified the oxygen dependence of therapeutic effects of isolated mitochondria. This reminds us of the application scenario of isolated mitochondria, where incorrect application may lead to serious consequences. It is generally believed that exogenous mitochondria have a relatively intact function and can replace the damaged mitochondria in the host cell [67]. Considering the oxygen dependence, we presume that the exogenous mitochondria are a load for the cell. In the presence of oxygen, the cell is able to handle this load and make it functional using the large amount of ATP produced by oxidative phosphorylation. However, in hypoxic conditions, host cells require additional energy to handle this load, which accelerates cell death. Also, according to our transcriptome data, the exogenous mitochondrial function is closely related to lipid metabolism, which may increase its oxygen dependence. In fact, there is little known about oxygen dependence, and this will be one of our future research directions.

The therapeutic effects of isolated mitochondria in our cell and animal models are similar to other studies [30, 31, 64, 65], which showed that mitochondrial intervention attenuated I/R injury, improved neurological outcomes, and reduced cerebral infarct size. Our data again confirmed the potential clinical application of mitochondrial transplantation. The transcriptomic data suggest that the therapeutic effect of mitochondria may be related to altered metabolism, especially lipid metabolism, providing clues for future mechanistic studies. Few studies focused on the behavior of isolated mitochondria *in vivo*, especially whether it can cross the brain-blood barrier. Nakamura et al. [65] injected mitochondria intravenously and found exogenous mitochondria distributed in the brain under ischemic-reperfusion condition. Shi et al. [36] injected isolated mitochondria intravenously in mice and found that the exogenous mitochondria distributed in various tissues including the brain, liver, kidney, muscle, and heart. However, we did not find that mitochondria can pass the intact blood-brain barrier in our projects. More research is needed on the permeability of the blood-brain barrier to mitochondria.

The discovery of mitochondrial component separation phenomenon was based on different mitochondrial labeling techniques, and this gives us a new perspective to study the behavior of mitochondria. To our knowledge, most studies [25, 30, 31, 33, 36, 64, 65] labeled mitochondria with a single MitoTracker dye or a fluorescent fusion protein and got a conclusion based on that. However, we used both techniques to label the same mitochondria. Surprisingly, we found the different markers are separated. Regardless of whether it was technical or not, at least, it proved that a different conclusion may be made based on a different single-label method. Therefore, previous works need to be revisited. This is one of the important information provided in this article. MitoTracker dyes are roughly divided into voltage-dependent and non-voltage-dependent. We chose the non-voltage-dependent dye MitoTracker Green to label mitochondria, which are covalently bound to the free sulfhydryl group of cysteine in mitochondrial protein [68, 69]. Therefore, the possibility of dye transfer between mitochondria is extremely low, and no studies have reported this dye-transfer phenomenon. Thus, MitoTracker Green represents the mitochondrial component that binds to it. Fluorescent fusion proteins (most fused with mitochondrial targeting sequence of cytochrome c oxidase subunit VIII) are another commonly used method of labeling mitochondria [33, 36, 37]. Due to the wide distribution in the mitochondria, both labeling methods can display mitochondria and are well overlapped (Figure 6(a)). When the extracted double-labeled mitochondria enter the host cell, the two markers are separated, which represents that different component (not subgroups) of mitochondria has different fates. Considering the lysosomal labeling and mitochondria dynamic protein Western blot experiments, it is indicated that the isolated mitochondria may function by fusing its useful part to the host mitochondria rather than replacing it entirely. The unfused part will enter the lysosome for degradation. Furthermore, we used iPSC to study the effect of cell connections on the entry of mitochondria into host cells. The result implied that tight intercellular connections will greatly reduce the red component of isolated mitochondria from entering the host cell.

Few studies have covered the fate of isolated mitochondria after being internalized by host cells. Cowan et al. [70] reported that after being incorporated into host cells, isolated mitochondria are transported to endosomes and lysosomes, and then, most of these mitochondria escape from the compartments and fuse with the endogenous mitochondrial network. Their work described the fate of exogenous mitochondria, treating mitochondria as a whole, while our work found that the different part of mitochondria has different fate, which is consistent with the interaction of components between intracellular membrane systems. This result reminds us to understand the behavior of mitochondria from a more microscopic perspective and to pay more attention to its communication with other organelles.

**Figure 10:**
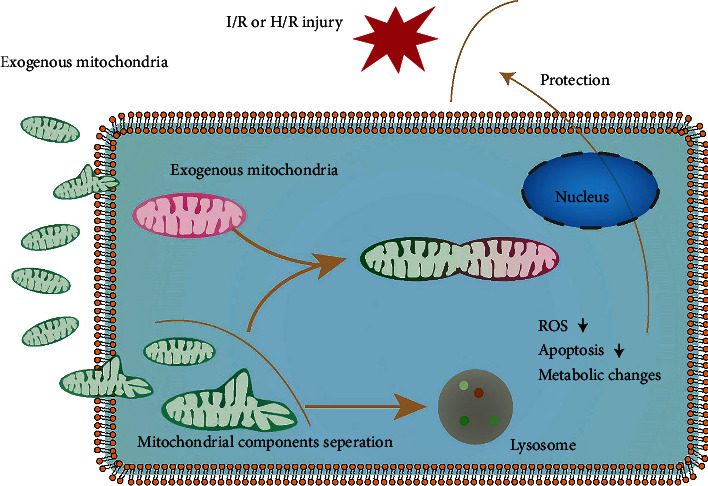
Hypothetical model diagram. When isolated exogenous enters host cells, the specific components of it undergo separation. Some fuses with the host mitochondria, while the other enters lysosome and undergoes lysosomal degradation. This process reduces ROS and apoptosis and alters metabolic profile, which in turn attenuates ischemia-reperfusion injury.

Based on our data, we hypothesized that when the exogenous mitochondria entered into the host cells, in the presence of oxygen, mitochondrial component separation occurred, reducing ROS levels, apoptosis, and altering cellular metabolism, thus improving cell survival (Figure 10). We made a reasonable assumption. Whether correct or not, it is of great significance, because this phenomenon will allow us to reexamine previous studies and consider this factor in future research design.

Still, there are limitations. The correctness of the conclusion is closely related to the mitochondrial label methods. When previous studies labeled mitochondria by a single method, they got incomplete information. The current label methods are mainly developed to display mitochondria in cells. Unpredictable events may happen when mitochondria are isolated. Therefore, a new fate tracking tool should be developed. Another limitation would be lacking proper controls in the fate experiments. Due to the superficial knowledge of the event, we had no idea where to intervene. Although we used a mitochondrial fission inhibitor, Mdivi-1, as a control (Figure S6), it did not seem to show any difference. Another similar study did not set up controls too [70]. Therefore, our future direction is to develop new mitochondrial label tools and clarify the fate and transportation of isolated mitochondria through proper controls. In addition, the mechanism conclusions need to be verified in vivo.

In general, studies focusing on mitochondrial transplantation therapy are in their infancy, but existing data indicate a promising clinical application. When there is no effective treatment for ischemia-reperfusion injury, mitochondrial transplantation therapy provides a new idea. However, more data is needed to confirm its safety and efficacy, and more mechanism studies are needed to figure out how it works. We hope our study provides useful information in this area and has enlightenment for future studies.

## 5. Conclusions

Mitochondrial transplantation may attenuate cerebral I/R injury. The mechanism may be related to selective mitochondrial component separation, altering cellular metabolism, reducing ROS, and apoptosis in an oxygen-dependent manner. The way of isolated mitochondrial transfer into the cell may be related to intercellular connection.

## Data Availability

The datasets generated during and/or analyzed during the current study are available from the author (xieqiang1990@gmail.com) on reasonable request.

## Abbreviations

ANOVA: Analysis of variance
ATP: Adenosine triphosphate
bFGF: Basic fibroblast growth factor
CCK-8: Cell Counting Kit-8
DAPI: 4′,6-Diamidino-2-phenylindole
DMEM: Dulbecco's modified Eagle's medium
ECA: External carotid artery
EGF: Epidermal growth factor
ESI: Electrospray ionization source
FCCP: Carbonyl cyanide 4-(trifluoromethoxy) phenylhydrazone)
H&E: Hematoxylin and eosin
H/R: Hypoxia-reoxygenation
HILIC: Hydrop interaction liquid chromatography
I/R: Ischemia-reperfusion
ICA: Internal carotid artery
iPSC: Pluripotent stem cell
KEGG: Kyoto Encyclopedia of Genes and Genomes
LC-MS: Liquid chromatography-mass spectrometry
MCA: Middle cerebral artery
MCAO: Middle cerebral artery occlusion
MMP/*ΔΨ*m: Mitochondrial membrane potential
mNSC: Mouse neural stem cell
mNSS: Modified neurological severity score
mtDNA: Mitochondrial DNA
N2a: Neuro-2a
OCR: Oxygen consumption rate
PBS: Phosphate buffer solution
PCA: Principal components analysis
PCR: Polymerase chain reaction
ROS: Reactive oxygen species
RNA-seq: RNA sequencing
SD: Standard deviation
TEM: Transmission electron microscopy
TTC: 2,3,5-Triphenyltetrazolium chloride
UPLC: Ultraperformance liquid chromatography
WB: Western blot.

## Additional Points

*Statement*. The presentation of the manuscript has been posted on Research Square [71].

## Supplementary Materials

Supplementary 1 Figure S1: mitochondrial morphology of N2a and mNSC. (A, B) Typical mitochondrial shape of N2a (A) and mNSC (B) labeled with MitoTracker™ Red CMXRos. (C, D) TEM image of mitochondrial shape in N2a (C) and mNSC (D). Red arrows indicate mitochondria.

Supplementary 2 Figure S2: culture and identification of mNSC. (A, B) mNSC suspension culture (A) and adherent culture (B). (C) Identification of mNSC by double-staining of Nestin and Sox2. Scale bar: 200 *μ*m and 50 *μ*m.

Supplementary 3 Figure S3: metabolic profile difference between mNSC and N2a. (A–D) Metabolomic data analysis of cationic mode; PCA (A) and heat map (B) illustrated the N2a and mNSC have different metabolic profile; the volcano plot showed 115 upregulated and 37 downregulated metabolites (C); bubble chart of enriched KEGG pathway showed the top 4 pathways were histidine metabolism (1), nicotinate and nicotinamide metabolism (2), alanine, aspartate, and glutamate metabolism (3), and D-glutamine and D-glutamate metabolism (4) (D). (E–H) Metabolomic data analysis of anion mode; PCA (E) and heat map (F); the volcano plot showed 108 upregulated and 24 downregulated metabolites (G); bubble chart of enriched KEGG pathway showed the top 4 pathways were alanine, aspartate, and glutamate metabolism (1), amino sugar and nucleotide sugar metabolism (2), fructose and mannose metabolism (3), and pentose phosphate pathway (4) (H).

Supplementary 4 Figure S4: tumorigenicity of mitochondria from mNSC and N2a. (A–G) N2a derived mitochondria tumorigenicity assay (*n* = 8); N2a-derived mitochondria were injected into the right axilla of nude mice and tested for tumorigenicity after 6 months; typical image of the nude mice (A), H&E staining of the injection site (B), heart (C), liver (D), spleen (E), lung (F), and kidney (G). (H–N) mNSC derived mitochondria tumorigenicity assay. Typical image of the nude mice (H), H&E staining of the injection site (I), heart (J), liver (K), spleen (L), lung (M), and kidney (N). Scale bar: 200 *μ*m.

Supplementary 5 Figure S5: the influence of the mitochondrial tag on cell and mitochondrial functionality. (A) Cell growth curve was detected by a CCK-8 assay between 293T (control) and 293T expressing COX8A gene N-terminal signal peptide-mCherry fusion protein (Mito-mCherry) (*n* = 6); (B) ROS levels of two cells detected by DCFH-DA probes through a fluorescence plate reader (*n* = 6), Rosup is a positive control drug; (C) ATP levels of two cells detected a luminescence plate reader (*n* = 6).

Supplementary 6 Figure S6: Mdivi-1 does not affect the process of mitochondrial component separation. Top, mitochondria of host 293T cell labeled with MitoBright Deep Red; isolated double-labeled mitochondria (MitoTracker Green and Mito-mCherry) were added to the medium of the host cell for 24 hours; bottom, the host cell was pretreated with 50 *μ*M Mdivi-1 for 6 hours and then cocultured with the double-labeled isolated mitochondria for 24 hours; scale bar: 10 *μ*m.

Supplementary 7 Table S1: related primer sequences for absolutely quantitative PCR analysis.

## References

[1] Gorelick P. B. (2019). The global burden of stroke: persistent and disabling. *Lancet Neurology*.

[2] GBD 2016 Lifetime Risk of Stroke Collaborators, Feigin V. L., Nguyen G. (2018). Global, regional, and country-specific lifetime risks of stroke, 1990 and 2016. *New England Journal of Medicine*.

[3] He Z., Ning N., Zhou Q., Khoshnam S. E., Farzaneh M. (2020). Mitochondria as a therapeutic target for ischemic stroke. *Free Radical Biology & Medicine*.

[4] Phipps M. S., Cronin C. A. (2020). Management of acute ischemic stroke. *BMJ*.

[5] Lapchak P. A., Zhang J. H. (2017). The high cost of stroke and stroke cytoprotection research. *Translational Stroke Research*.

[6] Yang Q., Huang Q., Hu Z., Tang X. (2019). Potential neuroprotective treatment of stroke: targeting excitotoxicity, oxidative stress, and inflammation. *Frontiers in Neuroscience*.

[7] Sekerdag E., Solaroglu I., Gursoy-Ozdemir Y. (2018). Cell death mechanisms in stroke and novel molecular and cellular treatment options. *Current Neuropharmacology*.

[8] Reis C., Wilkinson M., Reis H. (2017). A look into stem cell therapy: exploring the options for treatment of ischemic stroke. *Stem Cells International*.

[9] Patel R. A. G., McMullen P. W. (2017). Neuroprotection in the treatment of acute ischemic stroke. *Progress in Cardiovascular Diseases*.

[10] Zerna C., Thomalla G., Campbell B. C. V., Rha J. H., Hill M. D. (2018). Current practice and future directions in the diagnosis and acute treatment of ischaemic stroke. *Lancet*.

[11] Fernandez-Moriano C., Gonzalez-Burgos E., Gomez-Serranillos M. P. (2015). Mitochondria-targeted protective compounds in Parkinson’s and Alzheimer’s diseases. *Oxidative Medicine and Cellular Longevity*.

[12] Moskowitzova K., Orfany A., Liu K. (2020). Mitochondrial transplantation enhances murine lung viability and recovery after ischemia-reperfusion injury. *American Journal of Physiology. Lung Cellular and Molecular Physiology*.

[13] Chen H. H., Chen Y. T., Yang C. C. (2016). Melatonin pretreatment enhances the therapeutic effects of exogenous mitochondria against hepatic ischemia-reperfusion injury in rats through suppression of mitochondrial permeability transition. *Journal of Pineal Research*.

[14] Lin H. C., Liu S. Y., Lai H. S., Lai I. R. (2013). Isolated mitochondria infusion mitigates ischemia-reperfusion injury of the liver in rats. *Shock*.

[15] Blitzer D., Guariento A., Doulamis I. P. (2020). Delayed transplantation of autologous mitochondria for cardioprotection in a porcine model. *The Annals of Thoracic Surgery*.

[16] Guariento A., Blitzer D., Doulamis I. (2020). Preischemic autologous mitochondrial transplantation by intracoronary injection for myocardial protection. *The Journal of Thoracic and Cardiovascular Surgery*.

[17] Kaza A. K., Wamala I., Friehs I. (2017). Myocardial rescue with autologous mitochondrial transplantation in a porcine model of ischemia/reperfusion. *The Journal of Thoracic and Cardiovascular Surgery*.

[18] Masuzawa A., Black K. M., Pacak C. A. (2013). Transplantation of autologously derived mitochondria protects the heart from ischemia-reperfusion injury. *American Journal of Physiology. Heart and Circulatory Physiology*.

[19] McCully J. D., Cowan D. B., Pacak C. A., Toumpoulis I. K., Dayalan H., Levitsky S. (2009). Injection of isolated mitochondria during early reperfusion for cardioprotection. *American Journal of Physiology. Heart and Circulatory Physiology*.

[20] Orfany A., Arriola C. G., Doulamis I. P. (2020). Mitochondrial transplantation ameliorates acute limb ischemia. *Journal of Vascular Surgery*.

[21] Shi X., Bai H., Zhao M. (2018). Treatment of acetaminophen-induced liver injury with exogenous mitochondria in mice. *Translational Research*.

[22] Fu A., Shi X., Zhang H., Fu B. (2017). Mitotherapy for fatty liver by intravenous administration of exogenous mitochondria in male mice. *Frontiers in Pharmacology*.

[23] Elliott R. L., Jiang X. P., Head J. F. (2012). Mitochondria organelle transplantation: introduction of normal epithelial mitochondria into human cancer cells inhibits proliferation and increases drug sensitivity. *Breast Cancer Research and Treatment*.

[24] Jiang X. P., Elliott R. L., Head J. F. (2015). Exogenous normal mammary epithelial mitochondria suppress glycolytic metabolism and glucose uptake of human breast cancer cells. *Breast Cancer Research and Treatment*.

[25] Chang J. C., Chang H. S., Wu Y. C. (2019). Mitochondrial transplantation regulates antitumour activity, chemoresistance and mitochondrial dynamics in breast cancer. *Journal of Experimental & Clinical Cancer Research*.

[26] Huang T. H., Chung S. Y., Chua S. (2016). Effect of early administration of lower dose versus high dose of fresh mitochondria on reducing monocrotaline-induced pulmonary artery hypertension in rat. *American Journal of Translational Research*.

[27] Su Y., Zhu L., Yu X. (2016). Mitochondrial transplantation attenuates airway hyperresponsiveness by inhibition of cholinergic hyperactivity. *Theranostics*.

[28] Nakamura Y., Park J. H., Hayakawa K. (2020). Therapeutic use of extracellular mitochondria in CNS injury and disease. *Experimental Neurology*.

[29] Chang C. Y., Liang M. Z., Chen L. (2019). Current progress of mitochondrial transplantation that promotes neuronal regeneration. *Translational neurodegeneration*.

[30] Zhang Z., Ma Z., Yan C. (2019). Muscle-derived autologous mitochondrial transplantation: a novel strategy for treating cerebral ischemic injury. *Behavioural Brain Research*.

[31] Huang P. J., Kuo C. C., Lee H. C. (2016). Transferring xenogenic mitochondria provides neural protection against ischemic stress in ischemic rat brains. *Cell Transplantation*.

[32] Li H., Wang C., He T. (2019). Mitochondrial transfer from bone marrow mesenchymal stem cells to motor neurons in spinal cord injury rats via gap junction. *Theranostics*.

[33] Gollihue J. L., Patel S. P., Eldahan K. C. (2018). Effects of mitochondrial transplantation on bioenergetics, cellular incorporation, and functional recovery after spinal cord injury. *Journal of Neurotrauma*.

[34] Robicsek O., Ene H. M., Karry R. (2018). Isolated mitochondria transfer improves neuronal differentiation of schizophrenia-derived induced pluripotent stem cells and rescues deficits in a rat model of the disorder. *Schizophrenia Bulletin*.

[35] Wang Y., Ni J., Gao C. (2019). Mitochondrial transplantation attenuates lipopolysaccharide- induced depression-like behaviors. *Progress in Neuro-Psychopharmacology & Biological Psychiatry*.

[36] Shi X., Zhao M., Fu C., Fu A. (2017). Intravenous administration of mitochondria for treating experimental Parkinson's disease. *Mitochondrion*.

[37] Chang J. C., Wu S. L., Liu K. H. (2016). Allogeneic/xenogeneic transplantation of peptide-labeled mitochondria in Parkinson's disease: restoration of mitochondria functions and attenuation of 6-hydroxydopamine-induced neurotoxicity. *Translational Research*.

[38] Emani S. M., Piekarski B. L., Harrild D., del Nido P. J., McCully J. D. (2017). Autologous mitochondrial transplantation for dysfunction after ischemia- reperfusion injury. *The Journal of Thoracic and Cardiovascular Surgery*.

[39] Guariento A., Piekarski B. L., Doulamis I. P. (2021). Autologous mitochondrial transplantation for cardiogenic shock in pediatric patients following ischemia-reperfusion injury. *The Journal of Thoracic and Cardiovascular Surgery*.

[40] Ishikawa K., Toyama-Sorimachi N., Nakada K. (2010). The innate immune system in host mice targets cells with allogenic mitochondrial DNA. *The Journal of Experimental Medicine*.

[41] Zhang Q., Raoof M., Chen Y. (2010). Circulating mitochondrial DAMPs cause inflammatory responses to injury. *Nature*.

[42] Ramirez-Barbieri G., Moskowitzova K., Shin B. (2019). Alloreactivity and allorecognition of syngeneic and allogeneic mitochondria. *Mitochondrion*.

[43] Soares R., Ribeiro F. F., Lourenço D. M. (2020). Isolation and expansion of neurospheres from postnatal (P1−3) mouse neurogenic niches. *Journal of Visualized Experiments*.

[44] Rambold A. S., Kostelecky B., Elia N., Lippincott-Schwartz J. (2011). Tubular network formation protects mitochondria from autophagosomal degradation during nutrient starvation. *Proceedings of the National Academy of Sciences of the United States of America*.

[45] Sun C., Liu X., Wang B. (2019). Endocytosis-mediated mitochondrial transplantation: transferring normal human astrocytic mitochondria into glioma cells rescues aerobic respiration and enhances radiosensitivity. *Theranostics*.

[46] Zhao W., Xu Z., Cao J. (2019). Elamipretide (SS-31) improves mitochondrial dysfunction, synaptic and memory impairment induced by lipopolysaccharide in mice. *Journal of Neuroinflammation*.

[47] Augustyniak J., Lenart J., Zychowicz M., Stepien P. P., Buzanska L. (2017). Mitochondrial biogenesis and neural differentiation of human iPSC is modulated by idebenone in a developmental stage-dependent manner. *Biogerontology*.

[48] He M. D., Xu S. C., Lu Y. H. (2011). L-carnitine protects against nickel-induced neurotoxicity by maintaining mitochondrial function in Neuro-2a cells. *Toxicology and Applied Pharmacology*.

[49] Paliwal S., Chaudhuri R., Agrawal A., Mohanty S. (2018). Human tissue-specific MSCs demonstrate differential mitochondria transfer abilities that may determine their regenerative abilities. *Stem Cell Research & Therapy*.

[50] Boukelmoune N., Chiu G. S., Kavelaars A., Heijnen C. J. (2018). Mitochondrial transfer from mesenchymal stem cells to neural stem cells protects against the neurotoxic effects of cisplatin. *Acta Neuropathologica Communications*.

[51] Chen Q. F., Liu Y. Y., Pan C. S. (2018). Angioedema and hemorrhage after 4.5-hour tPA (tissue-type plasminogen activator) thrombolysis ameliorated by T541 via restoring brain microvascular integrity. *Stroke*.

[52] Zeng J., Chen Y., Ding R. (2017). Isoliquiritigenin alleviates early brain injury after experimental intracerebral hemorrhage via suppressing ROS- and/or NF-*κ*B-mediated NLRP3 inflammasome activation by promoting Nrf2 antioxidant pathway. *Journal of Neuroinflammation*.

[53] Ding R., Feng L., He L. (2015). Peroxynitrite decomposition catalyst prevents matrix metalloproteinase-9 activation and neurovascular injury after hemoglobin injection into the caudate nucleus of rats. *Neuroscience*.

[54] Yang M. Y., Yu Q. L., Huang Y. S., Yang G. (2019). Neuroprotective effects of andrographolide derivative CX-10 in transient focal ischemia in rat: Involvement of Nrf2/AE and TLR/NF-*κ*B signaling. *Pharmacological Research*.

[55] Cho D. Y., Jeun S. S. (2018). Combination therapy of human bone marrow-derived mesenchymal stem cells and minocycline improves neuronal function in a rat middle cerebral artery occlusion model. *Stem Cell Research & Therapy*.

[56] Pénzes M., Túrós D., Máthé D. (2020). Direct myosin-2 inhibition enhances cerebral perfusion resulting in functional improvement after ischemic stroke. *Theranostics*.

[57] Clark W. M., Lessov N., Dixon M., Eckenstein F. (1997). Monofilament intraluminal middle cerebral artery occlusion in the mouse. *Neurological Research*.

[58] Chen J., Sanberg P. R., Li Y. (2001). Intravenous administration of human umbilical cord blood reduces behavioral deficits after stroke in rats. *Stroke*.

[59] HAMM R. J., PIKE B. R., O'DELL D. M., LYETH B. G., JENKINS L. W. (1994). The rotarod test: an evaluation of its effectiveness in assessing motor deficits following traumatic brain injury. *Journal of Neurotrauma*.

[60] Alquisiras-Burgos I., Ortiz-Plata A., Franco-Pérez J., Millán A., Aguilera P. (2020). Resveratrol reduces cerebral edema through inhibition of _de novo_ SUR1 expression induced after focal ischemia. *Experimental Neurology*.

[61] Dock H., Theodorsson A., Theodorsson E. (2015). DNA methylation inhibitor Zebularine confers stroke protection in ischemic rats. *Translational Stroke Research*.

[62] Guo F., Yu X., Xu A. (2018). Japanese encephalitis virus induces apoptosis by inhibiting Foxo signaling pathway. *Veterinary Microbiology*.

[63] Baek A., Park E. J., Kim S. Y. (2018). High-frequency repetitive magnetic stimulation enhances the expression of brain-derived neurotrophic factor through activation of Ca2+–Calmodulin-Dependent protein kinase II-cAMP-response element-binding protein pathway. *Frontiers in Neurology*.

[64] Pourmohammadi-Bejarpasi Z., Roushandeh A. M., Saberi A. (2020). Mesenchymal stem cells-derived mitochondria transplantation mitigates I/R-induced injury, abolishes I/R-induced apoptosis, and restores motor function in acute ischemia stroke rat model. *Brain Research Bulletin*.

[65] Nakamura Y., Lo E. H., Hayakawa K. (2020). Placental mitochondria therapy for cerebral ischemia-reperfusion injury in mice. *Stroke*.

[66] Hayashida K., Takegawa R., Shoaib M. (2021). Mitochondrial transplantation therapy for ischemia reperfusion injury: a systematic review of animal and human studies. *Journal of Translational Medicine*.

[67] Lightowlers R. N., Chrzanowska-Lightowlers Z. M., Russell O. M. (2020). Mitochondrial transplantation-a possible therapeutic for mitochondrial dysfunction?: mitochondrial transfer is a potential cure for many diseases but proof of efficacy and safety is still lacking. *EMBO Reports*.

[68] Presley A. D., Fuller K. M., Arriaga E. A. (2003). MitoTracker Green labeling of mitochondrial proteins and their subsequent analysis by capillary electrophoresis with laser-induced fluorescence detection. *Journal of Chromatography. B, Analytical Technologies in the Biomedical and Life Sciences*.

[69] Chazotte B. (2011). Labeling mitochondria with MitoTracker dyes. *Cold Spring Harbor Protocols*.

[70] Cowan D. B., Yao R., Thedsanamoorthy J. K., Zurakowski D., del Nido P. J., McCully J. D. (2017). Transit and integration of extracellular mitochondria in human heart cells. *Scientific Reports*.

[71] Xie Q., Zeng J., Xu F. (2021). *Mitochondrial transplantation improves stroke-induced brain injury: possible involvement of selective component recombination*.

