# Supplementary figures and images for: Mitochondrial Transplantation Attenuates Cerebral Ischemia-Reperfusion Injury: Possible Involvement of Mitochondrial Component Separation
Source: Oxid Med Cell Longev. 2021 Nov 20;2021:1006636. doi: 10.1155/2021/1006636 (PMC8627565; doi:10.1155/2021/1006636)

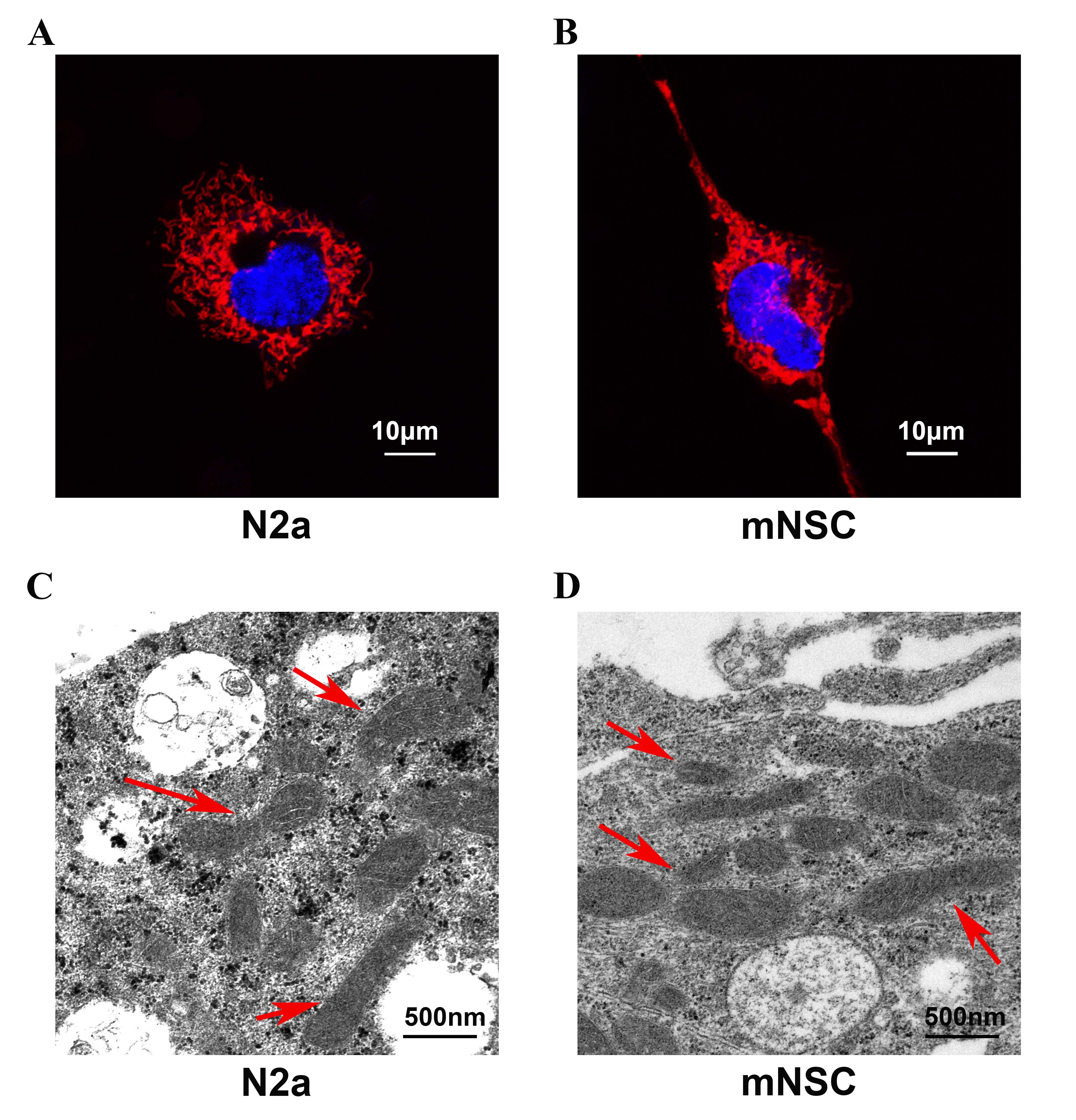

Supplement: Supplementary 1 — Figure S1: mitochondrial morphology of N2a and mNSC. (A, B) Typical mitochondrial shape of N2a (A) and mNSC (B) labeled with MitoTracker™ Red CMXRos. (C, D) TEM image of mitochondrial shape in N2a (C) and mNSC (D). Red arrows indicate mitochondria. [file 1006636.f1.jpg]

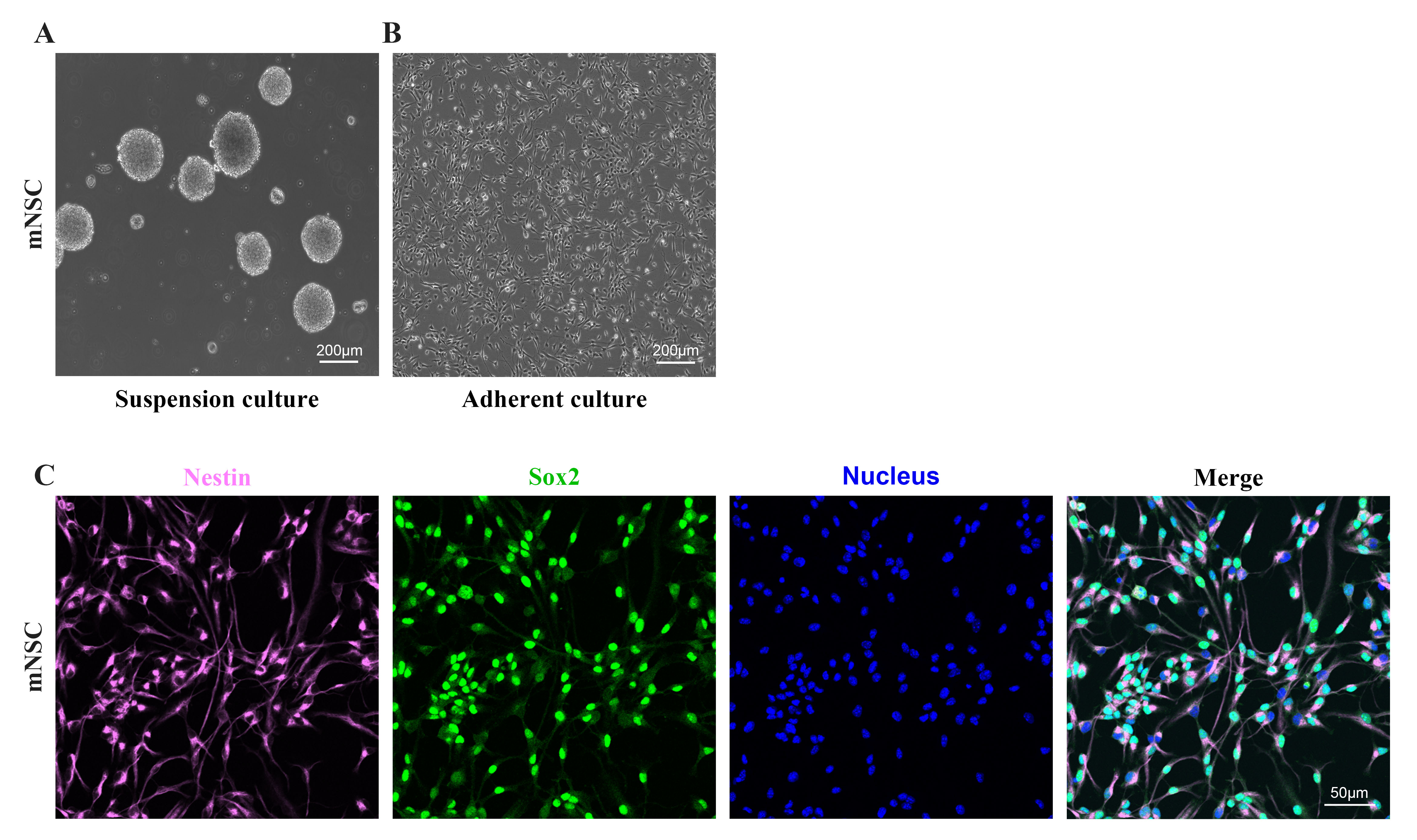

Supplement: Supplementary 2 — Figure S2: culture and identification of mNSC. (A, B) mNSC suspension culture (A) and adherent culture (B). (C) Identification of mNSC by double-staining of Nestin and Sox2. Scale bar: 200 μm and 50 μm. [file 1006636.f2.jpg]

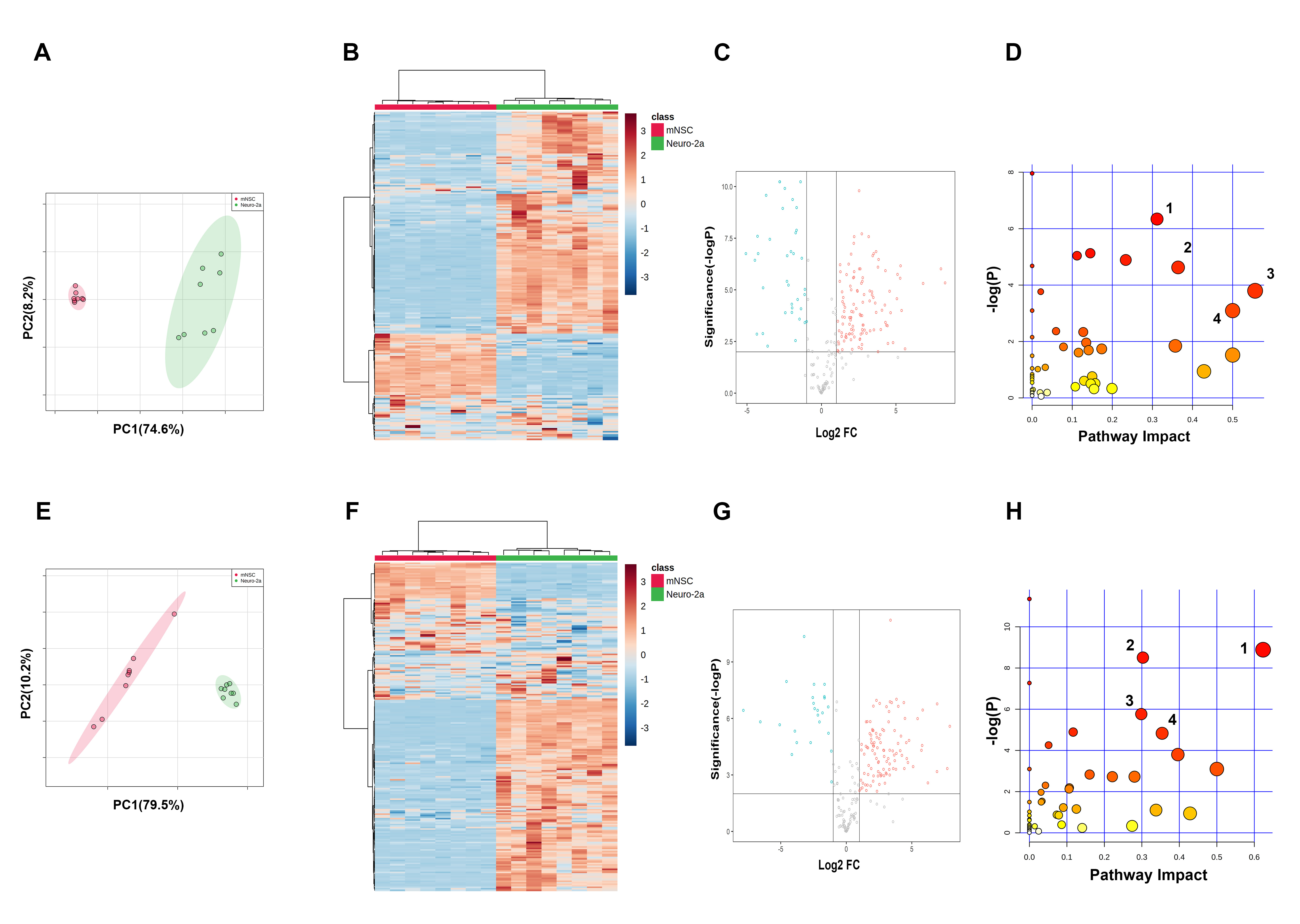

Supplement: Supplementary 3 — Figure S3: metabolic profile difference between mNSC and N2a. (A–D) Metabolomic data analysis of cationic mode; PCA (A) and heat map (B) illustrated the N2a and mNSC have different metabolic profile; the volcano plot showed 115 upregulated and 37 downregulated metabolites (C); bubble chart of enriched KEGG pathway showed the top 4 pathways were histidine metabolism (1), nicotinate and nicotinamide metabolism (2), alanine, aspartate, and glutamate metabolism (3), and D-glutamine and D-glutamate metabolism (4) (D). (E–H) Metabolomic data analysis of anion mode; PCA (E) and heat map (F); the volcano plot showed 108 upregulated and 24 downregulated metabolites (G); bubble chart of enriched KEGG pathway showed the top 4 pathways were alanine, aspartate, and glutamate metabolism (1), amino sugar and nucleotide sugar metabolism (2), fructose and mannose metabolism (3), and pentose phosphate pathway (4) (H). [file 1006636.f3.jpg]

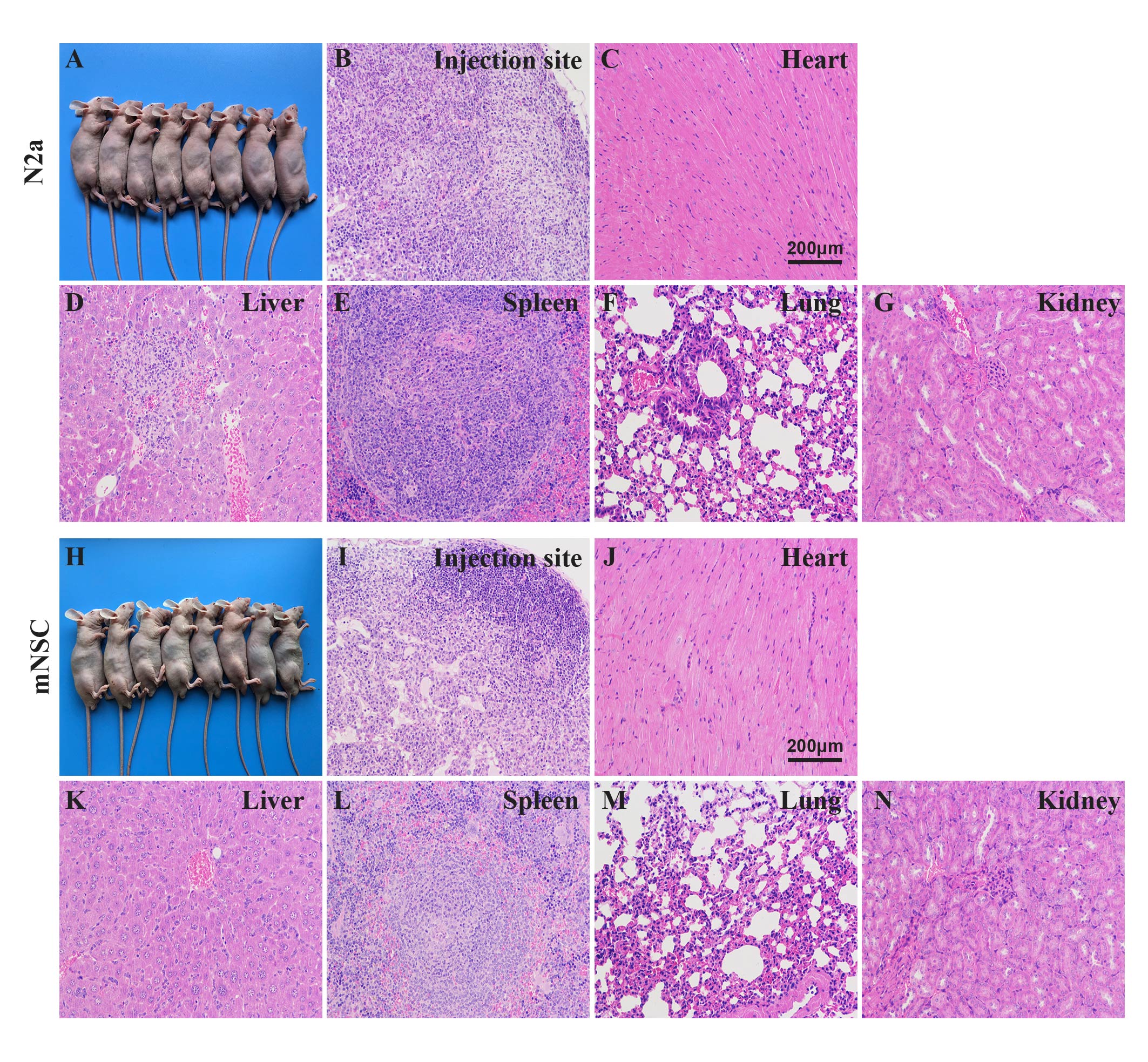

Supplement: Supplementary 4 — Figure S4: tumorigenicity of mitochondria from mNSC and N2a. (A–G) N2a derived mitochondria tumorigenicity assay (n = 8); N2a-derived mitochondria were injected into the right axilla of nude mice and tested for tumorigenicity after 6 months; typical image of the nude mice (A), H&E staining of the injection site (B), heart (C), liver (D), spleen (E), lung (F), and kidney (G). (H–N) mNSC derived mitochondria tumorigenicity assay. Typical image of the nude mice (H), H&E staining of the injection site (I), heart (J), liver (K), spleen (L), lung (M), and kidney (N). Scale bar: 200 μm. [file 1006636.f4.jpg]

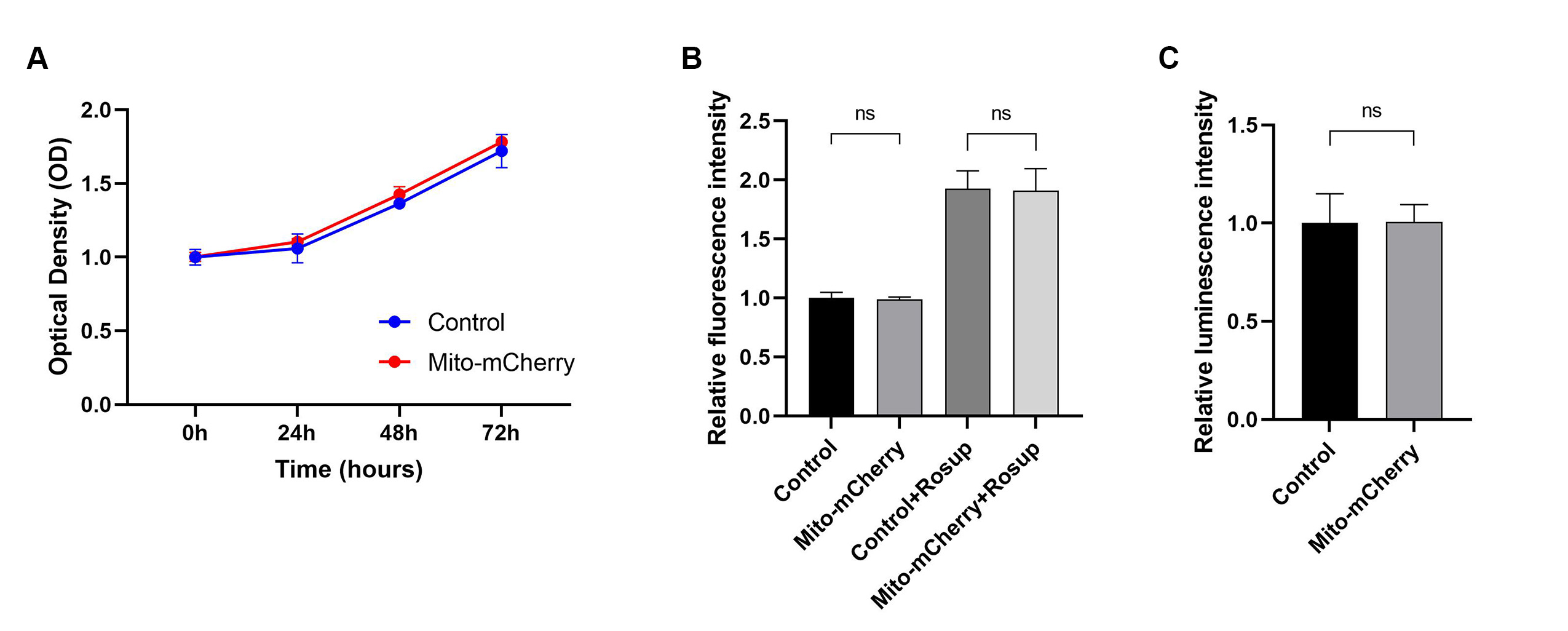

Supplement: Supplementary 5 — Figure S5: the influence of the mitochondrial tag on cell and mitochondrial functionality. (A) Cell growth curve was detected by a CCK-8 assay between 293T (control) and 293T expressing COX8A gene N-terminal signal peptide-mCherry fusion protein (Mito-mCherry) (n = 6); (B) ROS levels of two cells detected by DCFH-DA probes through a fluorescence plate reader (n = 6), Rosup is a positive control drug; (C) ATP levels of two cells detected a luminescence plate reader (n = 6). [file 1006636.f5.jpg]

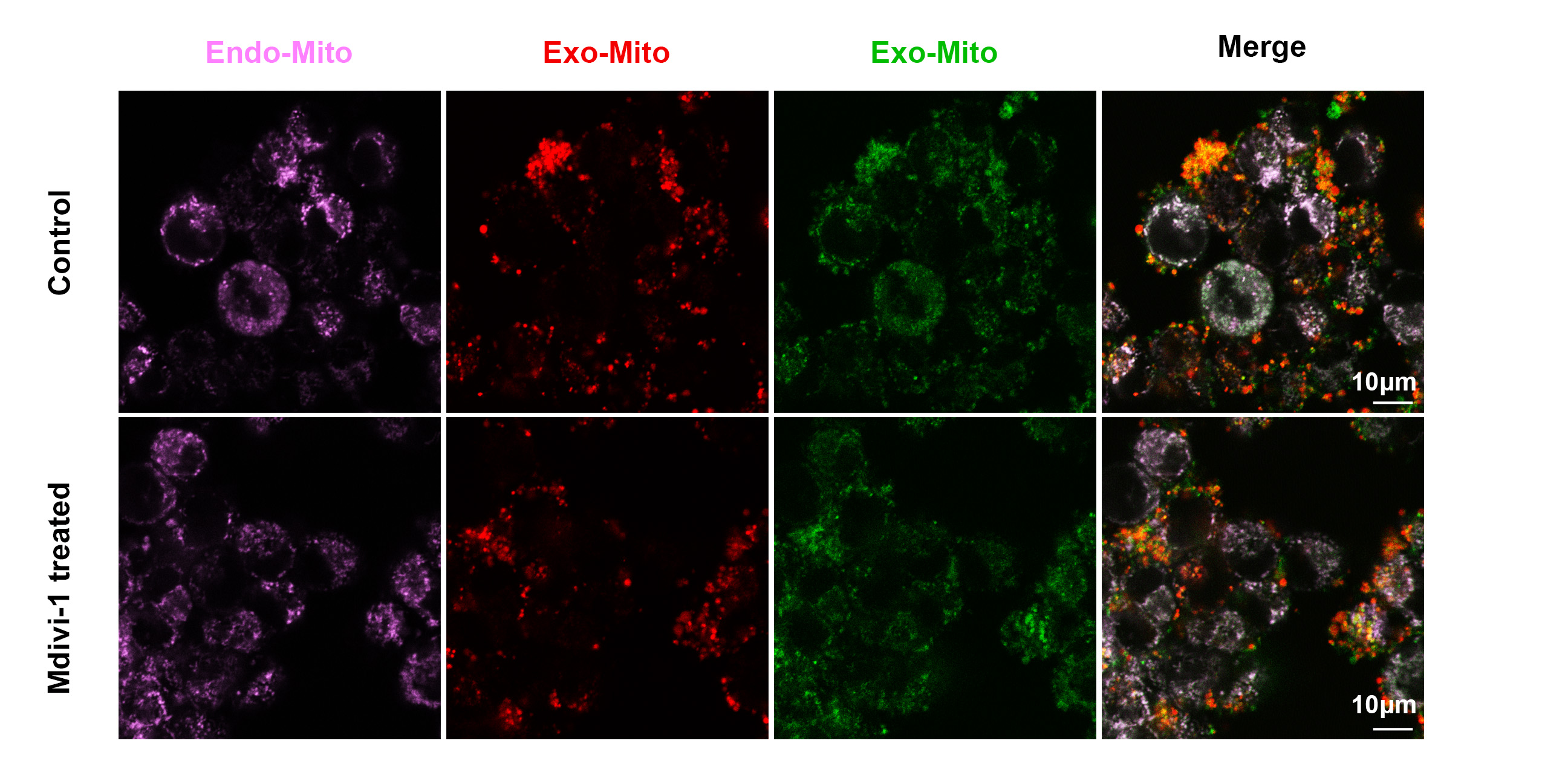

Supplement: Supplementary 6 — Figure S6: Mdivi-1 does not affect the process of mitochondrial component separation. Top, mitochondria of host 293T cell labeled with MitoBright Deep Red; isolated double-labeled mitochondria (MitoTracker Green and Mito-mCherry) were added to the medium of the host cell for 24 hours; bottom, the host cell was pretreated with 50 μM Mdivi-1 for 6 hours and then cocultured with the double-labeled isolated mitochondria for 24 hours; scale bar: 10 μm. [file 1006636.f6.jpg]
