# Supplementary material for: Mitochondrial Transplantation Attenuates Cerebral Ischemia-Reperfusion Injury: Possible Involvement of Mitochondrial Component Separation
Source: Oxid Med Cell Longev. 2021 Nov 20;2021:1006636. doi: 10.1155/2021/1006636 (PMC8627565; doi:10.1155/2021/1006636)
Supplement: Supplementary 7 — Table S1: related primer sequences for absolutely quantitative PCR analysis. [file 1006636.f7.docx]

**Table** **S1: Related primer sequences for absolutely quantitative PCR analysis**

| Genes | Forward primers (5′ - 3′) | Reverse primers (5′ - 3′) |
| --- | --- | --- |
| mt-RNR1 | AGCAATGAAGTACGCACACA | TTCCAAGCACACTTTCCAGT |
| mt-ND1 | GAGCTTTACGAGCCGTAGCC | CCCGGTTTGTTTCTGCTAGG |
| β-globin | GACACACAACCCCAGAAACA | GCCTCACCACCAACTTCATC |
| β-actin | TGGAATCCTGTGGCATCCAT | GCTAGGAGCCAGAGCAGTAA |
